# Use of jejunal serosal patch and pyloric exclusion in the management of complex duodenal injury

**DOI:** 10.1308/rcsann.2023.0074

**Published:** 2024-03-06

**Authors:** D Alsaadi, D Low, A Osman, M Mcmonagle

**Affiliations:** ^1^ University Hospital Waterford, Ireland; ^2^ University of Pennsylvania, USA; ^3^ Saint Luke’s General Hospital, Kilkenny, Ireland; ^4^ Imperial College Healthcare NHS Trust, UK

**Keywords:** Duodenal injury, Pyloric exclusion, Jejunal serosal patch, Trauma, Damage control surgery

## Abstract

**Background:**

Duodenal injuries are relatively rare but remain a management challenge with a high incidence of postoperative complications. Guidelines from the World Society of Emergency Surgery and American Association for the Surgery of Trauma favour a primary repair for less-complex injuries, but the management of more complex duodenal trauma remains controversial with varying techniques supported, including pyloric exclusion, omental or jejunal patch closure, gastrojejunostomy and pancreatoduodenectomy. We describe the techniques used in one case of complex duodenal trauma.

**Technique:**

The duodenum is approached via a standard laparotomy with Kocherisation. Primary repair of the duodenal perforations is performed using a 3/0 polydioxanone suture (PDS), followed by mobilisation of a loop of mid-jejunum against the area of duodenal trauma over the primary repair as a jejunal serosal patch. The antimesenteric jejunal serosal border is sutured to the serosa of the duodenum (serosa only) using a 3/0 PDS. Pyloric exclusion is then performed through an anterior gastrostomy, to control the volume of gastric juice entering the duodenum. The pylorus is sutured closed using an absorbable suture followed by closure of the anterior gastrostomy using a GIA stapling device.

## Background

**Figure 1:**
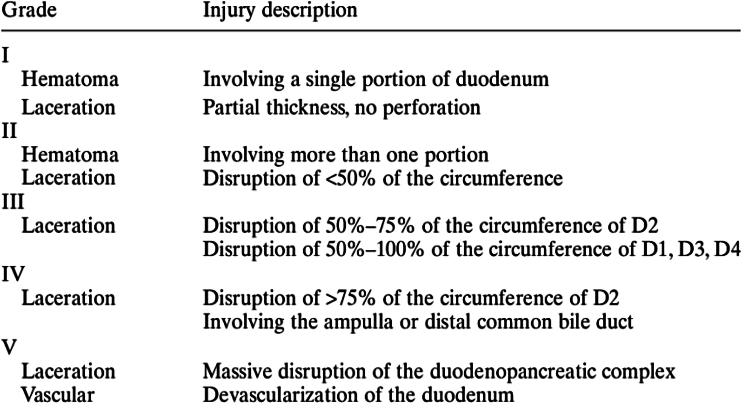
American Association for the Surgery of Trauma classification (from Moore and Jones^2^ with permission)

Duodenal injuries are relatively rare owing to their well-protected retroperitoneal position, but remain challenging to both diagnose and treat, with a high incidence of postoperative complications. The lack of a complete serosal envelope in addition to the high volume of fluid passing daily into and through the duodenum, place it at higher risk of leakage, failure of repair and fistula formation. In addition, because of the forces involved, there is a high incidence of additional injuries, in particular pancreatic trauma.^1^ Owing to the lack of robust level I data, expert consensus dictates managing simple perforations (American Association for the Surgery of Trauma grade I–II; Figure 1) with primary repair alone. For more complex injuries, especially after blunt trauma where the injury has a tendency to evolve over a number of days with delayed leakage of repair (grade III–V), management remains divided.^3–5^ One technique described is the jejunal serosal patch, which reinforces the primary repair by offering an additional layer of protection^6^ to mitigate against leakage. Pyloric exclusion is another technique described after severe duodenal injury, as a temporary measure to prevent fluids entering the lumen of the duodenum from the stomach, thereby enabling primary repair without resection and, in theory, reducing leakage from the primary repair.^3,4^ Following a high-velocity road traffic accident, a healthy 62-year-old man sustained multiple abdominal and bony injuries. Intraperitoneal and retroperitoneal free air with fat stranding around the second part of duodenum was noted on computed tomography trauma imaging. Damage control laparotomy findings included two duodenal perforations, an ischaemic terminal ileum, significant retroperitoneal contamination and parenchymal pancreatic injury with some minor bleeding from superior mesenteric branches and small pancreatitis vessels. This paper describes the management and techniques used to treat the duodenal injuries.

## Technique

**Figure 2:**
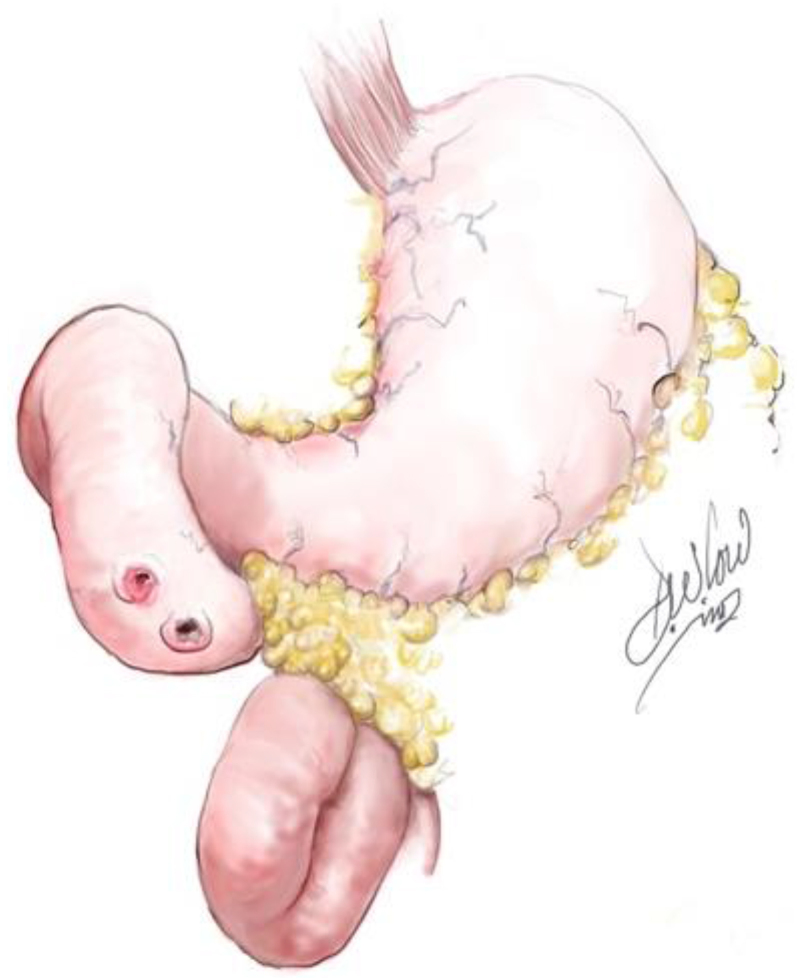
Duodenal perforations

The abdomen is prepared as standard, and a midline incision performed for a trauma laparotomy to immediately control bleeding and contamination. The duodenum is explored as part of the trauma laparotomy and mobilised using a right medial visceral rotation (Cattell–Braasch manoeuvre) and Kocherisation. In our case, multiple traumatic perforations of the duodenum are noted, including grade III injury (Figure 2).

### Primary repair

**Figure 3:**
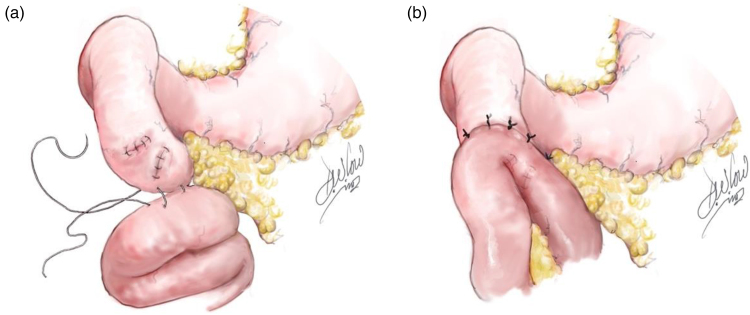
(a) Primary repair of duodenal perforations and jejunal serosal patch (inferior border). (b) Jejunal serosal patch (superior border)

A primary, single-layer repair was performed using a 3/0 polydioxanone suture (PDS; Ethicon) (Figure 3) and drains were inserted adjacent to the duodenum. A standard trauma laparotomy was completed and the abdomen was packed, but a temporary closure device (Abthera) placed with a planned take back once physiology was restored. The patient was then transferred to the intensive care unit for restoration of physiology, including reversal of the coagulopathy, hypothermia and acidosis.

### Jejunal serosal patch

At the relook laparotomy, leakage was noted from the blunt duodenal perforation areas throughout segments 3 and 4 of a very friable duodenum with bruising to the head of pancreas. To manage further maturing dehiscence, a loop of mid-jejunum was mobilised tension-free and its antimesenteric border placed against the primary repair to reinforce it. The healthy jejunal serosa was sutured directly to the duodenum using 3/0 PDS simple interrupted tension-free sutures, superior and inferior to the area of duodenal injury as a serosal patch (Figure 3). The pancreatic parenchymal injury was gently debrided. The pancreatic duct was not involved (clinically and at follow-up magnetic resonance cholangiopancreatography). Drains were placed behind, in front of, above and below the duodenal–pancreatic injury to control any fistulae or contamination that may develop.

### Pyloric exclusion

**Figure 4:**
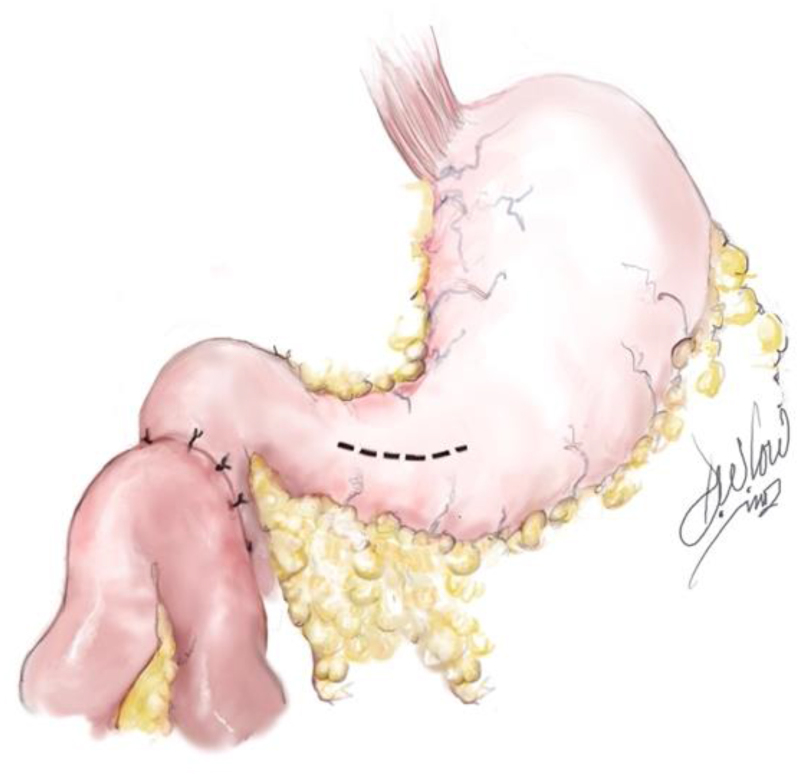
Anterior gastrotomy

**Figure 5:**
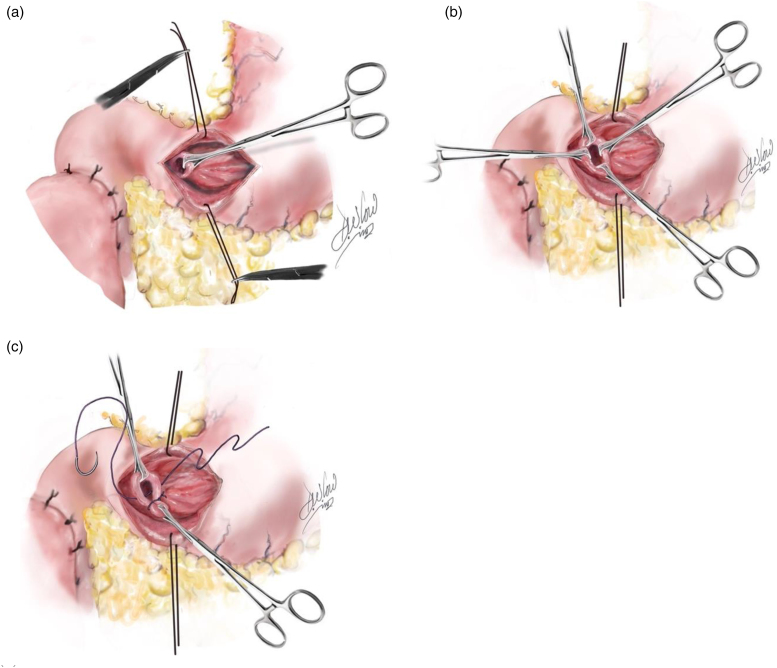
Pyloric exclusion: (a) identification of pylorus, (b) lifting of pylrous, (c) ligation of pylorus

To control gastric fluid in-flow into the duodenum, an anterior gastrotomy was created to access the pylorus (Figure 4). Anchoring sutures were placed on either side of the gastrostomy to aid exposure. The pylorus was identified and grasped using Babcock forceps to elevate it into the surgical field for suturing. The pylorus was then closed using interrupted simple absorbable sutures (3/0 vicryl; Ethicon), thereby creating complete gastric outflow obstruction (Figure 5). The anterior gastrotomy was then closed using a GIA stapler and the suture line buried using 3/0 PDS. With regards to feeding, an open (surgical) jejunostomy tube was placed during this procedure to enable gut feeding. In addition, a nasogastric tube was inserted to allow for gastric drainage and feeding at a later stage.

## Discussion

To date, the literature supports the need for more complex procedures when dealing with more complex injuries.^3–5^ Extensive duodenal injuries with complete devascularisation and pancreatic head injuries often require an emergency pancreaticoduodenectomy.^7–9^ The techniques described here have been proven to protect the primary repair and reduce complications including suture line dehiscence. However, choosing when to utilise these techniques remains a dilemma faced by trauma surgeons owing to a lack of standardisation and clear indications. Currently, the surgeon must consider and alter treatment based on the patient’s situation because performing these techniques does prolong the operative period, which is not always possible in complex trauma patients in whom duodenal damage is unlikely to be an isolated injury. Excluding the pylorus allows the duodenum to rest temporarily by decreasing the fluid volume, diverting gastric contents and avoiding activation of pancreatic enzymes. The pylorus will re-open following pyloric exclusion; the mean time to opening varies based on type of suture used, being approximately 2–3 and 4–6 weeks with absorbable and non-absorbable sutures, respectively.^10^ Jejunal serosal patch is commonly used in the treatment of duodenal ulcer perforation, where it has eliminated the need for synthetic material and aids by providing a layer of protection.^6^ Research supports the use of pyloric exclusion and jejunal serosal patch in select complex cases with a reduction in morbidity noted, but queries the benefit of its use in simpler cases because it may prolong hospital stay and confer no survival or outcome benefit.^11–13^ There are multiple ways to establish nutrition following duodenal trauma and pyloric exclusion. The primary surgeon opted to utilise open jejunostomy in this particular case to keep the mucosa healthy by feeding it, in turn reducing critical care complications.^2^ Another viable option is total parental nutrition, but this is associated with numerous additional problems.^14^ Feeding in our patient was introduced gradually; enterally through the nasogastric tube on day 9 and then orally on day 23. Bilious output was noted consistently from day 13 indicating pylorus opening. The patient had an uncomplicated postoperative course and before discharge on day 53 the nasogastric and jejunostomy tubes were removed at the bedside. The patient was discharged home without any surgical or gastrointestinal complications to date.

## References

[1] Sharma AK. Management of pancreaticoduodenal injuries. *Indian J Surg* 2012; **74**: 35. 23372305 10.1007/s12262-011-0386-3 PMC3259167

[2] Moore EE, Jones TN. Benefits of immediate jejunostomy feeding after major abdominal trauma—a prospective, randomized study. *J Trauma Acute Care Surg* 1986; **26**: 874–881. 10.1097/00005373-198610000-00003 3095557

[3] Velmahos GC, Constantinou C, Kasotakis G. Safety of repair for severe duodenal injuries. *World J Surg* 2008; **32**: 7–12. 17952703 10.1007/s00268-007-9255-4

[4] Fraga GP, Biazotto G, Bortoto JB *et al.* The use of pyloric exclusion for treating duodenal trauma: case series. *Sao Paulo Med J* 2008; **126**: 337–341. 19274322 10.1590/S1516-31802008000600009 PMC11025995

[5] Ordoñez CA, Parra MW, Millán M *et al.* Damage control in penetrating duodenal trauma: less is better – the sequel. *Colomb Med (Cali)* 2021; **52**: e4104509. 34188326 10.25100/cm.v52i2.4509 PMC8216054

[6] Bekele A, Kassa S, Taye M. The jejunal serosal patch procedure: a successful technique for managing difficult peptic ulcer perforation. *East Centr Afr J Surg* 2016; **21**: 63–67.

[7] Ivatury RR. Duodenal injuries: small but lethal lesions. *Cir Gen* 2003; **25**: 59–65.

[8] Foley WJ, Gaines RD, Fry WJ. Pancreaticoduodenectomy for severe trauma to the head of the pancreas and the associated structures: report of three cases. *Ann Surg* 1969; **170**: 759. 5347554 10.1097/00000658-196911000-00007 PMC1387654

[9] De Kerpel W, Hendrickx T, Vanrykel J-P *et al.* Whipple procedure after blunt abdominal trauma. *J Trauma Acute Care Surg* 2002; **53**: 780–783. 10.1097/00005373-200210000-00028 12394884

[10] DeSantis M, Devereux DF, Thompson D. Pyloric exclusion. suture material of choice. *Am Surg* 1987; **53**: 711–714. 2827549

[11] Dubose JJ, Inaba K, Teixeira PG *et al.* Pyloric exclusion in the treatment of severe duodenal injuries: results from the national trauma data bank. *Am Surg* 2008; **74**: 925–929. 18942615 10.1177/000313480807401009

[12] Park YC, Kim HS, Kim DW *et al.* Time from injury to initial operation may be the sole risk factor for postoperative leakage in AAST-OIS 2 and 3 traumatic duodenal injury: A retrospective cohort study. *Medicina* 2022; **58**: 801. 35744064 10.3390/medicina58060801 PMC9229050

[13] Critselis AN, Papaioannou AN. Serosal patch vs duodenojejunostomy in duodenal stump closure. *Arch Surg* 1977; **112**: 670. 856110 10.1001/archsurg.1977.01370050130029

[14] Rhee P, Hadjizacharia P, Trankiem C *et al.* What happened to total parenteral nutrition? The disappearance of its use in a trauma intensive care unit. *J Trauma* 2007; **63**: 1215–1222. 18212641 10.1097/TA.0b013e31815b83e9

